# Identification and Quantification of Stilbenes (Piceatannol and Resveratrol) in *Passiflora edulis* By-Products

**DOI:** 10.3390/ph13040073

**Published:** 2020-04-20

**Authors:** Karolline Krambeck, Ana Oliveira, Delfim Santos, Maria Manuela Pintado, João Baptista Silva, José Manuel Sousa Lobo, Maria Helena Amaral

**Affiliations:** 1 UCIBIO-REQUIMTE, MedTech, Laboratory of Pharmaceutical Technology, Department of Drug Sciences, Faculty of Pharmacy, University of Porto, Rua de Jorge Viterbo Ferreira, 228, 4050 313 Porto, Portugal; dsantos@ff.up.pt (D.S.); slobo@ff.up.pt (J.M.S.L.); hamaral@ff.up.pt (M.H.A.); 2 CBQF–Centre for Biotechnology and Fine Chemistry, Faculty of Biotechnology, Catholic University of Portugal, Rua Diogo Botelho 1327, 4169-005 Porto, Portugal; alsoliveira84@gmail.com (A.O.); mpintado@porto.ucp.pt (M.M.P.); 3 Department of Geosciences, University of Aveiro, Campus of Santiago, 3810 193 Aveiro, Portugal; madeirarochas@netmadeira.com

**Keywords:** stilbenes, *Passiflora edulis*, by-products, piceatannol, resveratrol

## Abstract

Recently, studies on the by-products from the food industry, such as passion fruit seeds, have significantly increased, as these can have an added value, due to their properties, such as potential antioxidant activity. This study was conducted to determine the presence of piceatannol and resveratrol in various extracts of passion fruit (*Passiflora edulis*) seeds from Madeira Island and a commercial passion fruit oil was used as reference. The commercial oil and the extracts that were obtained by traditional Soxhlet method with ethanol and acetone did not reveal the presence of the two stilbenes, piceatannol and resveratrol. However, the extracts that were obtained by the ultrasound method showed significant amounts of piceatannol and resveratrol when compared with the commercial oil. The presence of these compounds indicates that this oil could have potential application in cosmetic and pharmaceutical industries, due to their proven antioxidant and anti-aging properties.

## 1. Introduction

Madeira Island is a Portuguese territory that is located in the Atlantic Ocean, which has a temperate tropical climate, which allows for the cultivation of various species of passion fruit. The purple passion fruit (*Passiflora edulis*) is one of the species used in the production of juices by the food industry. Only the passion fruit pulp is used in the production of the juice, and the discarded seeds generate thousands of tons of waste every year [1,2,3]. The generation of waste has high costs in its treatment and, based on this, the use of this waste in other processes that can produce value-added products results in great interest for the society and scientific community [4,5,6].

It is mentioned in the literature that the purple passion fruit seeds oil has antioxidant, anti-inflammatory, and skin lightening effect, among others [7,8,9]. The oil is rich in stilbenes, vitamins, and catechin. It is described in the literature the presence of piceatannol and resveratrol in passion fruit from Japan and Brazil [10].

Several studies highlight the effectiveness of resveratrol due to its antioxidant activity, anti-aging potential, neuroprotective effect, and anti-cancer properties, particularly in cases of leukemia, and in cancers of the breast and colon [11,12,13,14,15,16,17,18,19].

The benefits of piceatannol have not been studied as extensively as in the case of resveratrol [20]. Piceatannol (3,3′,4′,5-trans-tetrahydroxystilbene) is a polyphenolic compound that has been found in some plants, including grapes, passion fruit, white tea, rhubarb, peanuts, berries, and some mushroom species [10,21,22].

Stilbenes are compounds that are considered to be phytoalexins, because they protect plants against fungi and toxins. The presence of an additional hydroxyl group in the piceatannol structure gives it greater antioxidant activity when compared to its prodrug, resveratrol [21,23]. Piceatannol also promotes collagen production, preventing skin damage and inhibiting melanin synthesis [24].

Yokozawa and Kim studies [25] have shown that piceatannol has a better inhibitory activity of the tyrosinase enzyme, as well as decreases melanin production, better than resveratrol and kojic acid, a potent skin whitening agent. 

In previous studies, we evaluated the antioxidant activity of passion fruit seeds extracts, which were obtained by two methods, Soxhlet and Ultrasound, using various solvents. Extracts that were obtained by both methods using ethanol and acetone were chosen, since they showed the highest antioxidant capacity [8].

In view of the abundance of passion fruit waste as by-products in Portugal, sustainable management of these by-products is necessary. In this context, the objective of this work was to identify and quantify stilbenes, piceatannol, and resveratrol, by High Performance Liquid Chromatography (HPLC), in *Passiflora edulis* seeds oil from Madeira Island and compare with commercial passion fruit seeds oil, in order to evaluate the potential of these antioxidant compounds for further applications by the pharmaceutical and cosmetic industries. 

## 2. Results and Discussion

### 2.1. Standard Samples

**Figure 1:**
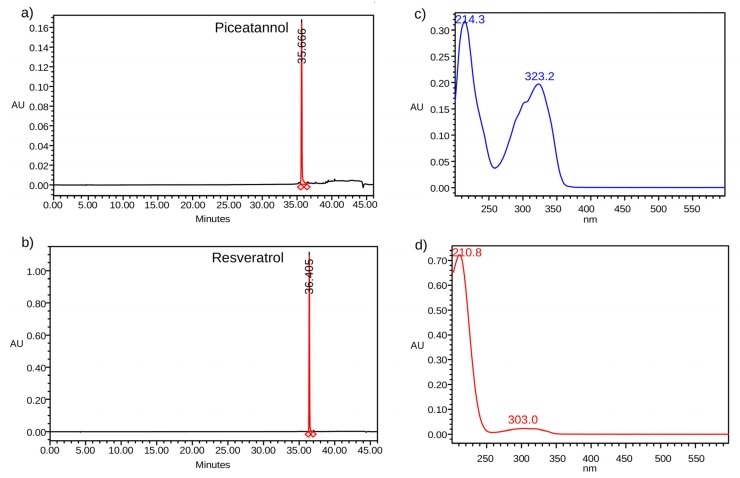
High-performance liquid chromatography with diode array detection (HPLC-DAD) chromatograms of the standard solutions: (**a**) piceatannol; (**b**) resveratrol. UV spectra of the standard solutions: (**c**) piceatannol; and, (**d**) resveratrol.

Using the RP-HPLC method, piceatannol and resveratrol peaks were detected with retention times of 35.66 and 36.40 min., respectively. Figure 1 shows the chromatograms and UV spectra of piceatannol and resveratrol. The identification of both stilbenes was confirmed with this HPLC-DAD. 

Two calibration curves, one for piceatannol and the other for resveratrol, were obtained with R^2^ = 0.999 and R^2^ = 0.996, respectively.

### 2.2. Soxhlet Extraction

There was no evidence of the presence of piceatannol and resveratrol in both extracts that were obtained by the Soxhlet method. The chromatograms showed unknown peaks, although some of the peaks obtained the same retention time, they did not absorb at the same UV wavelength as piceatannol and resveratrol. Because piceatannol and resveratrol are sensitive to high temperatures, these compounds may have been degraded during the eight-hour extraction of the Soxhlet method. Although the Soxhlet is a method with excellent extraction performance, in the case of polyphenols, due to the solvent heating at boiling temperatures for several hours, the degradation of phenolic compounds might occur [26].

Similar results have recently been described by Viganó and collaborators [27]. The study described by these authors demonstrated that the amount of piceatannol in the extracts of passion fruit bagasse obtained by the Soxhlet method was lower than the amount of this stilbene in the extracts that were obtained by maceration and extraction by pressurized liquid (PLE).

### 2.3. Ultrasound Extraction

**Figure 2:**
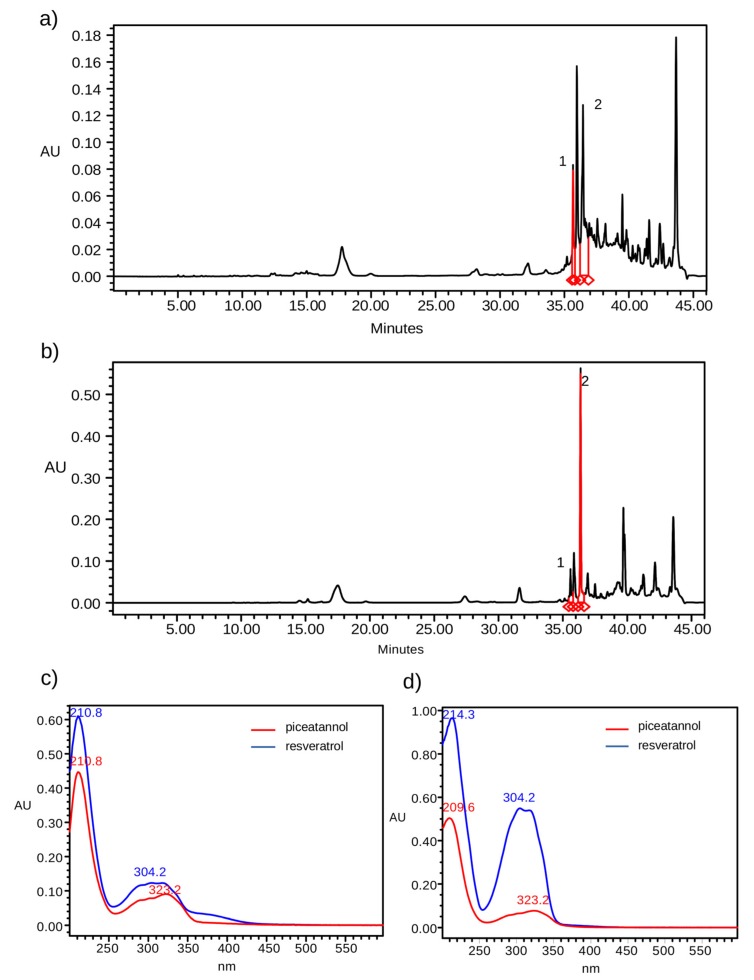
HPLC-DAD chromatograms (320 nm) of the extract obtained by the ultrasound method with ethanol (**a**) and with acetone (**b**). Number 1 and 2 corresponds to piceatannol and resveratrol, respectively. The UV spectra of piceatannol (red) and resveratrol (blue) detected on ethanol (**c**) and acetone (**d**) extracts.

The HPLC-DAD method allowed for separating piceatannol and resveratrol in a single run, as can be seen in the Figure 2. In HPLC chromatograms two peaks were identified as piceatannol and resveratrol by comparison of the ultraviolet (UV) spectra and retention time.

**Figure 3:**
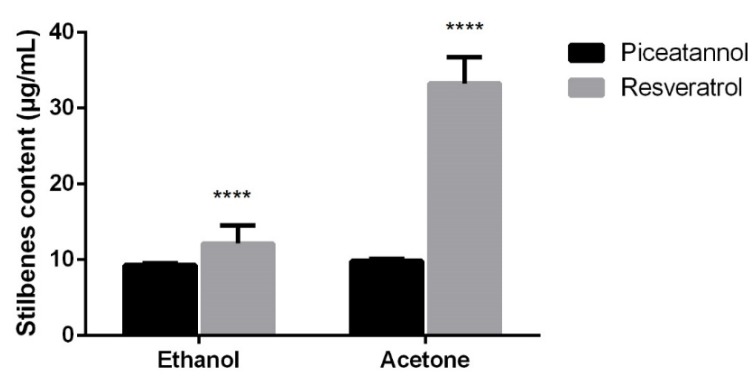
Piceatannol and resveratrol content (μg/mL) in ethanol and acetone extracts obtained with ultrasound method. Results are expressed as Mean ± SD. Statistical comparisons were made using one-way ANOVA, followed by the Tukey’s multiple comparisons test. Values significantly different from piceatannol (**** *p* < 0.05).

There are no significant differences regarding the amounts of piceatannol in the samples obtained with ethanol and acetone, according to Figure 3. However, there is a higher amount of resveratrol in samples extracted with acetone. When comparing the amount of piceatannol and resveratrol found in the extracts, it was verified that the extracts obtained using ethanol showed small differences of stilbenes content, whereas in the case of extracts that were obtained with acetone, the amount of resveratrol was significantly higher than the amount of piceatannol. These results corroborate other previous studies, in which the amount of resveratrol was higher than the amount of piceatannol found in plants [28,29,30]. Some authors showed that the amount of resveratrol in grapes was approximately four times higher than that of piceatannol (0.78 μg/g and 3.18 μg/g, respectively) [31].

Ultrasound extraction is a widely used method, since it is low cost, simple, and generally presents better results than conventional extraction methods [32]. This improvement in efficiency might be justified, because ultrasound is based on the energy of sound waves, promoting a good penetration of the solvent into the sample, thus increasing the contact surface as well as the acoustic cavitation produced, facilitating the release of contents [33,34].

A general overview of the results that were obtained by other authors allows for concluding that the ultrasound method is faster, more efficient, and more selective for polyphenols than the Soxhlet method [35].

A recent study investigated and quantified the polyphenol content in *Passiflora subpeltata* pulp from India by UPHLC-MS analysis. Significant amounts of epicatechin, ferulic acid, and protocatechuic acid were detected [36]. Rimando and collaborators detected up to 422 ng/g of piceatannol in species of blueberries from Mississippi, North Carolina. Significant amounts of resveratrol were also found in these fruits [37].

### 2.4. Commercial Oil

**Figure 4:**
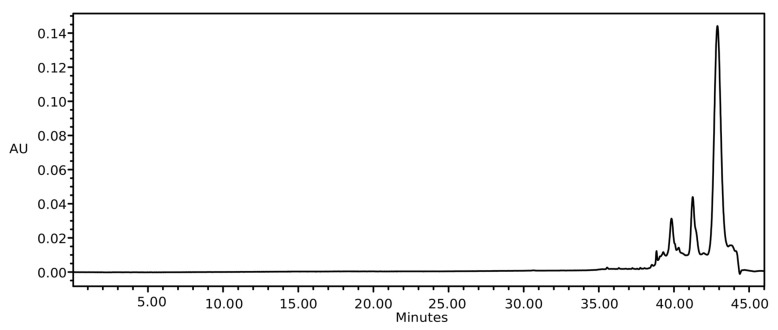
Chromatograms of the commercial passion fruit oil.

In Figure 4, it can be observed that the commercial passion fruit oil had no piceatannol or resveratrol. However, there were other unknown peaks.

In a previous study, the same commercial oil presented lower antioxidant activity in relation to the extracts that were obtained by ultrasound using acetone and ethanol as solvents. This activity was determined while using DPPH (2,2-diphenyl-1-picryl-hidrazil) and ABTS (2,2’-azino-bis-(3-ethylbenzthiazoline-6-sulfonic acid) methods [8]. Despite obtaining antioxidant activity, it might be due to the presence of many other compounds of the passion fruit oil, such as vitamin c and gallic acid.

## 3. Materials and Methods

### 3.1. Samples Preparation

Passion fruit seeds were obtained from the food industry of Madeira Island. These seeds were then dried in a stove and, after that, the extracts were prepared. These extracts were prepared according to Krambeck and collaborators [8]. The extracts were prepared using ethanol and acetone, with two preparation methods: Soxhlet and ultrasound.

### 3.2. Chemicals and Standards

Piceatannol and resveratrol standards, as well as ethanol and formic acid, were obtained from Sigma Aldrich (London, UK). Acetone was purchased from Fisher Chemical (Loughborough, UK). Methanol was purchased from VWR Chemicals (Vila Nova de Gaia, PT).

The stock solutions containing 1mg/mL of piceatannol and the same concentration for resveratrol in ethanol were prepared. All of the solutions were stored at −4 °C. Subsequently, for the calibration curve, standard solutions with concentrations ranging from 1.25–20 µg/mL for piceatannol and 0.625–35 µg/mL for resveratrol were prepared.

### 3.3. Methods

**Figure 5:**
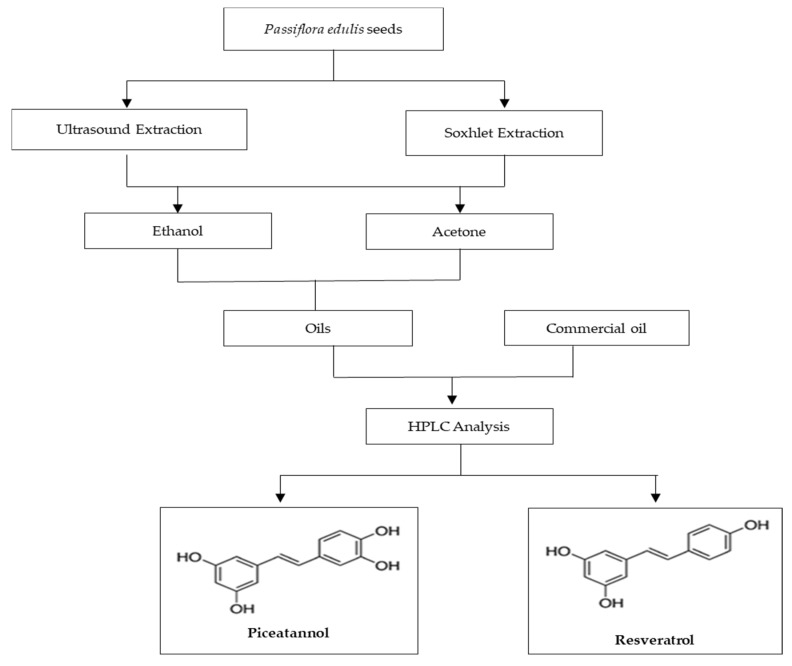
Flow diagram for the extraction and separation by HPLC of piceatannol and resveratrol.

The flow diagram for the extraction of the two stilbenes, piceatannol and resveratrol, can be seen in Figure 5. Briefly, after preparing the extracts, these were compared with a commercial oil from Akoma (London, UK), regarding the content of the stilbenes studied through HPLC analysis. In addition to determining the presence of stilbenes, the content of these elements in the extracts were also quantified. 

For the extracts that were obtained by Soxhlet, each selected solvent was heated to its boiling point, and the reflux was maintained for eight hours. For the extracts that were obtained by ultrasound, an ultrasound bath (35 kHz/80 W) (Sonorex RK100h, Bandelin, Germany) was used. The extraction time was 60 min at room temperature. At the end of all tests, the solvents were removed while using a rotary vacuum evaporator R-300 (Buchi, Flawil, Switzerland).

#### Reverse Phase High-Performance Liquid Chromatography (RP-HPLC)

RP-HPLC was carried out with some modifications, according to Lai and collaborators [38], for the simultaneous determination of two polyphenols: piceatannol and resveratrol. Analyses were carried out using a high-performance liquid chromatography (HPLC) Waters 2690 Separations Module, with photodiode array detector (PDA-Waters 996), and a 20 µL aliquot of the extract was injected onto a Waters ACE Equivalence C18 column (250 × 4.6 mm i.d.; 5 µm particle size). The mobile phases consisted of (A) water with 0.1% formic acid and (B) methanol with 0.1% formic acid. The total run time was 46 min., being 0–10 min., 0–15% B; 10–20 min., 15% B; 20–30 min., 15–35% B; 30–35 min., 35–100% B; 35–40 min., 100% B; 40–41 min., 100–0% B; 41–46 min., 0% B. All of the measurements were carried out at a flow rate of 0.8 mL/min., using a wavelength of 320nm. Peaks corresponding to piceatannol and resveratrol were analyzed by comparison with the retention times and UV spectras of their respective standard solutions and then quantified through calibration curves. The results were expressed in µg/mL oil. All of the analyses were carried out in triplicate. 

### 3.4. Statistical Analysis

The results were statistically evaluated by one-way analysis of variance (ANOVA), in which significant differences at the 5% level were analyzed by the Tukey’s test. SPSS Software (Version statistic 26, IBM SPSS, Chicago, IL, USA) was used for the statistical analysis in this study.

## 4. Conclusions

In this study, resveratrol and piceatannol were not detected either in the extracts of by-products of *Passiflora edulis* that were obtained by the Soxhlet method or in the commercial oil.

Extracts obtained by the ultrasound method using ethanol or acetone showed significant amounts of stilbenes such as piceatannol and resveratrol. Passion fruit by-products can be used in cosmetic and pharmaceutical industries having an added value, in addition to reducing the environmental pollution, avoiding the burning or landfill of waste.

The obtained results also suggest the possibility of production of *Passiflora edulis* seeds oil with green solvents and the potential interest of this product to industries, as it represents a low-cost ingredient.
